# Conserved Transcriptional Regulatory Programs Underlying Rice and Barley Germination

**DOI:** 10.1371/journal.pone.0087261

**Published:** 2014-02-18

**Authors:** Li Lin, Shulan Tian, Shawn Kaeppler, Zongrang Liu, Yong-Qiang (Charles) An

**Affiliations:** 1 USDA-ARS, Plant Genetics Research Unit, Donald Danforth Plant Sciences Center, Saint Louis, Missouri, United States of America; 2 Department of Plant Pathology, University of Wisconsin, Madison, Wisconsin, United States of America; 3 Department of Agronomy, University of Wisconsin, Wisconsin, United States of America; 4 USDA-ARS, Appalachian Fruit Research Station, Kearneysville, West Virginia, United States of America; University of Massachusetts, United States of America

## Abstract

Germination is a biological process important to plant development and agricultural production. Barley and rice diverged 50 million years ago, but share a similar germination process. To gain insight into the conservation of their underlying gene regulatory programs, we compared transcriptomes of barley and rice at start, middle and end points of germination, and revealed that germination regulated barley and rice genes (BRs) diverged significantly in expression patterns and/or protein sequences. However, BRs with higher protein sequence similarity tended to have more conserved expression patterns. We identified and characterized 316 sets of conserved barley and rice genes (cBRs) with high similarity in both protein sequences and expression patterns, and provided a comprehensive depiction of the transcriptional regulatory program conserved in barley and rice germination at gene, pathway and systems levels. The cBRs encoded proteins involved in a variety of biological pathways and had a wide range of expression patterns. The cBRs encoding key regulatory components in signaling pathways often had diverse expression patterns. Early germination up-regulation of cell wall metabolic pathway and peroxidases, and late germination up-regulation of chromatin structure and remodeling pathways were conserved in both barley and rice. Protein sequence and expression pattern of a gene change quickly if it is not subjected to a functional constraint. Preserving germination-regulated expression patterns and protein sequences of those cBRs for 50 million years strongly suggests that the cBRs are functionally significant and equivalent in germination, and contribute to the ancient characteristics of germination preserved in barley and rice. The functional significance and equivalence of the cBR genes predicted here can serve as a foundation to further characterize their biological functions and facilitate bridging rice and barley germination research with greater confidence.

## Introduction

Seed germination is a biological process important to plant development, plant evolution and agricultural production. Strictly defined, germination begins with the uptake of water by dry quiescent seeds and ends with visible emergence of an embryo tissue from its surrounding tissues [1]. Seed germination is accompanied by many distinct metabolic, cellular and physiological changes. For example, upon imbibition, the dry quiescent seeds take up water and rapidly resume many fundamental metabolic activities such as respiration, RNA metabolism, and protein synthesis using surviving structures and components in the desiccated cells. These concerted biological activities transform a dehydrated and resting embryo with almost undetectable metabolism into one with vigorous metabolism culminating in growth [2], [3].

Transcriptional regulatory program underlying seed germination and its associated biological pathways were investigated in divergent plant species [4], [5], [6], [7], [8], [9], [10], [11]. Extremely complex transcriptional regulatory programs are activated over the course of seed germination. In barley germination and seedling growth, 50% of examined genes are expressed in dry and germinating seeds at a detectable level. Twenty-five percent of those examined genes are differentially regulated over the course of seed germination and seedling growth. Based on global and dynamic expression changes of the germination-regulated genes, the transcriptional regulatory program underlying barley seed germination is divided into early and late phases. Each phase is accompanied by differential expression of a distinct set of genes and biological pathways. For example, the early phase of seed germination is accompanied by transcriptional up-regulation of cell wall synthesis and regulatory components including transcription factors, signaling proteins, and post-translational modification proteins. During the late germination phase, histone families and many metabolic pathways are up-regulated. Stress related pathways and seed storage protein genes are down-regulated through the entire course of germination. Comparing transcriptomes of barley and *Arabidopsis* showed that high accumulation of many seed stored transcripts in *Arabidopsis* and barley dry seeds have been preserved for 200 million years of monocot-dicot divergence [9], [11].

Barley and rice have been divergent for 50 million years, but share a great similarity in seed germination and seedling growth [3], [12]. For example, both rice and barley are endospermic and starch cereal species, and have a highly conserved seed storage mobilization pathway. Both rice and barley produce hydrolytic enzymes in aleurone tissues during seed germination and seedling growth, and translocate the hydrolytic enzymes to starch endosperm for mobilizing seed storage reserves. Seed germination and its associated production of hydrolytic enzymes are induced by gibberellic acid through a highly conserved transduction pathway [10], [13], [14], [15]. To gain an insight into transcriptional regulatory programs underlying the conserved characteristics of barley and rice germination, we determined transcriptomes of rice grains at start-, mid- and end-germination points, and developed a bioinformatic and evolutionary approach to compare them with our previously determined transcriptome of barley at the equivalent germination stages [9]. Genome-wide sequence comparison identified germination regulated rice and barley gene pairs with a strong sequence similarity. While a small percentage of these pairs showed similar expression patterns over the course of seed germination, a majority had divergent expression pattern. The analysis also identified a collection of germination regulated barley-rice gene sets. The rice and barley genes in each set shared strong similarities in protein sequences and expression patterns. Gene expression patterns and protein sequences changes quickly if there are no functional constraints [16], [17], [18], [19], [20], [21], [22]. Seed germination is accomplished through concerted activities of many gene products, which are mainly defined by their protein sequences and accumulation patterns. The preservation of germination-regulated expression patterns and protein sequences of the barley and rice genes in each set suggests that the barley and rice genes were functionally important and equivalent in germination, and likely contributed to the molecular and cellular processes conserved in barley and rice germination.

## Results

### Transcriptomes of Barley and Rice at Three Distinct and Equivalent Developmental Stages of Germination

An objective of this study was to compare transcriptomes of rice and barley over the course of germination and to identify germination regulated barley and rice genes with conserved protein sequences and expression patterns. Since expression of germination related genes are often differentially regulated with respects to specific developmental stages over the course of seed germination [6], [9], it is critical to compare their transcript accumulation levels at distinct and equivalent physiological stages. Our previous studies showed that transcriptional regulatory program underlying seed germination is divided into early and late germination phases that are separated by the mid-time point of germination [9]. Transcriptomes of barley at start- (dry), middle- (9 hr) and end-points of germination (18 hr) were previously determined and used for the comparison [9]. It took 42 hours for radicles to emerge from rice grains at the germination condition identical to barley germination. To compare transcriptomes of germinating rice and barley grains at their equivalent stages of barley germination, we examined transcriptomes of rice at 0 (dry), 21 and 42 hours of germination as start-, middle- and end-stages of germination. Three independent biological replications were conducted for each stage in rice and barley transcriptome assays.

Both barley and rice transcriptome data used in this study were produced using the Affymetrix GeneChip technologies (GeneChip Barley Genome Array and GeneChip Rice Genome Array), and were analyzed using identical statistical approaches and parameters to reduce variation from different transcriptome assay platforms and statistical analysis. One-way ANOVA identified a total of 3599 barley and 18665 rice probe-sets that were differentially regulated between any two examined stages of germination with a false discovery rate less than 5%. Considering the potential that non-specific hybridization between paralogous genes could cause an inaccurate assignment of signal intensity to gene family members, the probe-sets flagged by Affymetrix as potentially cross-hybridizing probes were removed from further analysis. A total of 2537 barley and 13813 rice probe sets were identified as germination regulated genes, and were used for further comparative analysis. A much higher number of germination regulated probe-sets were identified in rice than in barley. It was partially caused by the fact that the GeneChip Rice Genome Array has two times as many probe-sets as the GeneChip Barley Genome Array. In addition, probe-sets on barley array were designed using EST sequences while the ones on the rice array were designed using genes predicted from genome sequence, which are likely to lead to a lower percentage of germination regulated genes on the barley array than on the rice array.

### Conservation and Divergence of Transcriptional Regulatory Programs Underlying Barley and Rice Germination

**Figure 1:**
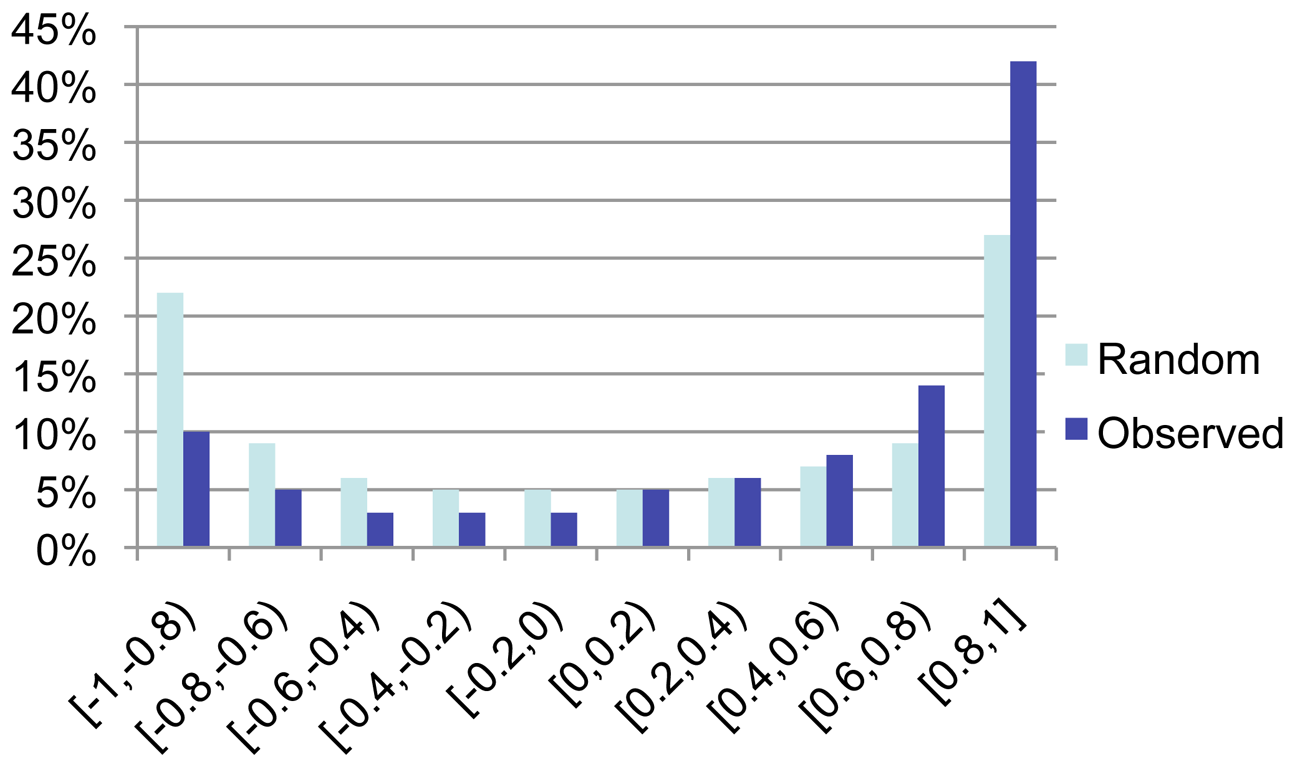
Distribution of Pearson Correlation Co-efficiency between Expression Patterns of Barley and Rice Genes. The germination regulated barley and rice genes (BRs) were paired randomly and paired based on their sequence similarity with an e-value less than −50 respectively; and their PCC values were determined. The distribution of PCC value for BR genes with e-value less than −50 (dark blue) were compared with randomly paired BR genes (light blue). The percentage of BRs (Y-axis) in each defined PCC value range (X-axis) was graphed.

A total of 1507 pairs of barley and rice genes (BRs) with protein sequence similarity at an e-value less than −50 were identified among the germination regulated barley and rice genes. The BRs contained 805 barley and 1054 rice genes (Table 1). Pearson correlation coefficients (PCC) between log2 signal intensities of each paired barley and rice genes at start-, mid- and end-stages of germination were calculated to determine the similarity of their expression patterns. Sixty percent of the BRs had a PCC value higher than 0.5, indicating that the barley and rice genes in each of the BRs had a good similarity in their transcript accumulation patterns (Figure 1, Table 2). However, forty percent of the BRs had PPC value lower than 0.5, indicating that a significant percentage of BRs had low similarity or no similarity in their expression patterns. Thus, the BRs with high protein sequence similarity preferentially preserved their expression patterns after rice and barley diverged from their most recent ancestor. However, a significant percentage of the BRs had evolved into different gene expression patterns.

**Table 1:** Summary of Germination Regulated BRs and cBRs.

No. of BRs with an e-value less than −50	1507	
Species	Barley	Rice
**No. of Distinct Genes**	805	1054

No. of BRs with PCC >0.9 and e-value < = −50	483	
Species	Barley	Rice
**No. of Distinct Genes**	368	388
**in Single-gene cBRs**	288	358
**in multi-gene cBRs**	80	30
**Single-gene/Distinct**	78.26%	92.27%

**Table 2:** Relationship Between Protein Sequence Similarity and Expression Similarity of Barley and Rice Genes.

Sequence Similarity\PCC value	[−1,−0.5)	[−0.5,0)	[0,0.5)	[0.5,1]	p value
**BRs with e-value < = −100**	18%	8%	14%	60%	< = 0.01
**BRs with e-value from −50 to −100**	16%	9%	16%	59%	< = 0.01
**BRs with e-value from −20 to−50**	23%	12%	12%	54%	< = 0.1
**BRs with e-value from −5 to −20**	36%	15%	12%	37%	< = 1
**random**	38%	13%	12%	37%	

A collection of randomly paired barley/rice genes were generated from the germination regulated barley and rice genes. The randomly paired BRs had a relatively symmetrical distribution of PCC value with a slightly higher percentage at a range of PCC value from 0.8 to 1.0 than that from −0.8 to −1.0. Interestingly, twenty-seven percent of the randomly paired BRs had a PCC value greater than 0.8 (Figure 1).

Percentage of BRs with similar expression patterns (PCC value from 0.5 to 1.0) positively correlated with their protein sequence similarities in the e value range of −5 to −100 (Table 2). However, there was little difference in distribution of PCC values between BRs with e value ranging from −50 to −100 and BRs with e value less than −100. Chi-square analysis was performed to compare distributions of PCC values between randomly paired BRs and BRs with a given range of e value. There was a significant difference in distribution of PCC values between BRs with e value from −50 to −100 and randomly paired BRs at P<0.01 (Table 2). However, there was no significant difference in distribution of PCC values between BR genes with e value from −20 to −50 and random paired BRs at P value of 0.1. Thus, the BRs at e-values less than −50 were used for identification of BRs that had conserved expression patterns.

### Barley and Rice Genes with Conserved Protein Sequences and Germination Regulated Expression Patterns (cBRs)

A total of 483 BRs with a PCC value higher than 0.9 were identified among the 1507 germination regulated BR genes. Those BRs accounted for 32% of the germination regulated BRs. The 483 BRs were comprised of 368 distinct barley genes and 388 distinct rice genes. Those genes represented a small percentage of the 2537 barley and 13813 rice germination regulated genes. Thus, majority of the germination-regulated genes had diverged beyond our thresholds in protein sequences, gene expression patterns or in both. The 483 BRs were further merged into 262 single-gene cBRs containing only one gene from each species and 60 multi-gene cBRs (Table 1 and Table 3). Barley and rice genes in each of those BRs were differentially regulated during seed germination, and shared strong similarity in both protein sequences and transcriptional expression patterns. We referred to the BRs as conserved BRs (cBRs). Each multi-gene cBR had at least three genes with one-to-many, many-to-one and many-to-many barley and rice gene relationship. Any pair of “orthologous” or paralogous genes in each multi-gene cBR had sequence similarity with an e-value less than −50 and expression pattern similarity with a PCC value higher than 0.9. The largest multi-gene cBR (cBR_M2) encoded a U-box domain containing RING protein family and had a total of 20 rice and barley genes (Table 3). However, the numbers of rice and barley genes in each cBRs were not always equally distributed. For example, the cBR_M2 was composed of 17 barley genes and 3 rice RING protein genes.

**Table 3:** The cBRs and Their Expression Patterns and Functions.

cBR ID	No. of barley genes	No. of rice genes	No. of total genes	Expression Patterns	Early Phase	Late Phase	MapMan Functional Groups	Gene Annotation
cBR_1	1	1	2	4	1.2	8.1	RNA.regulation of transcription.Silencing Group	anti-silencing protein 1,
cBR_2	1	1	2	6	−1.5	3.0	not assigned.unknown	expressed protein
cBR_3	1	1	2	1	1.7	24.5	DNA.synthesis/chromatin structure	replication protein A 70 kDa DNA-binding subunit,
cBR_4	1	1	2	3	2.8	−2.2	RNA.processing.ribonucleases	CCR4-NOT transcription complex subunit 7,
cBR_5	1	1	2	4	1.1	12.4	not assigned.unknown	NO_MATCH
cBR_6	1	1	2	4	1.1	6.6	not assigned.unknown	NO_MATCH
cBR_7	1	1	2	3	3.9	−2.0	protein.postranslational modification.kinase.receptor like cytoplasmatic kinase VII	protein kinase,
cBR_8	1	1	2	5	−1.3	−1.7	misc.acid and other phosphatases	hydrolase/protein serine/threonine phosphatase,
cBR_9	1	1	2	2	9.4	−1.1	protein.degradation.subtilases	subtilisin-like protease precursor,
cBR_10	1	1	2	8	−2.0	−4.1	TCA/org. transformation.TCA.pyruvate DH.E1	pyruvate dehydrogenase E1 component alpha subunit
cBR_11	1	1	2	3	7.4	−1.4	misc.UDP glucosyl and glucoronyl transferases	transferase, transferring glycosyl groups,
cBR_12	1	1	2	1	2.9	3.0	amino acid metabolism.synthesis.aspartate family.threonine.threonine synthase	threonine synthase, chloroplast precursor,
cBR_13	1	1	2	1	1.7	1.9	protein.degradation.ubiquitin.ubiquitin	polyubiquitin 2,
cBR_14	1	1	2	8	−3.1	−6.4	development.late embryogenesis abundant	late embryogenesis abundant protein D-34,
cBR_15	1	1	2	5	1.2	−3.2	development.unspecified	expressed protein
cBR_16	1	1	2	5	1.0	−2.4	not assigned.unknown	NO_MATCH
cBR_17	1	1	2	3	2.1	−2.4	signalling.calcium	grancalcin,
cBR_18	1	1	2	8	−1.5	−2.6	not assigned.unknown	early fruit mRNA,
cBR_19	1	1	2	3	5.3	−2.2	stress.abiotic.cold	SRC2,
cBR_20	1	1	2	1	1.5	2.9	not assigned.unknown	NO_MATCH
cBR_21	1	1	2	8	−2.6	−1.6	protein.degradation.autophagy	autophagy-related protein 8 precursor,
cBR_22	1	1	2	1	2.2	1.7	not assigned.unknown	nucleolar protein,Nop52 containing protein, expressed
cBR_23	1	1	2	4	1.2	8.8	misc.peroxidases	peroxidase 1 precursor,
cBR_24	1	1	2	8	−2.1	−1.8	not assigned.no ontology	monoglyceride lipase,
cBR_25	1	1	2	8	−1.5	−2.1	stress.biotic	lectin precursor,
cBR_26	1	1	2	8	−2.2	−1.4	not assigned.unknown	expressed protein
cBR_27	1	1	2	4	1.0	2.0	protein.synthesis.misc ribosomal protein	60S ribosomal protein L13a-2,
cBR_28	1	1	2	7	−2.6	−1.1	not assigned.unknown	NO_MATCH
cBR_29	1	1	2	8	−1.9	−2.4	not assigned.no ontology	lipid binding protein,
cBR_30	1	1	2	1	5.0	3.1	RNA.regulation of transcription.unclassified	aspartic proteinase nepenthesin-1 precursor,
cBR_31	1	1	2	8	−1.6	−4.3	stress.abiotic.heat	heat shock protein 82,
cBR_32	1	1	2	3	16.6	−5.1	signalling.in sugar and nutrient physiology	phi-1-like phosphate-induced protein,
cBR_33	1	1	2	1	1.5	1.4	protein.targeting.chloroplast	signal peptidase I-1,
cBR_34	1	1	2	6	−2.1	7.4	misc.short chain dehydrogenase/reductase (SDR)	estradiol 17-beta-dehydrogenase 8,
cBR_35	1	1	2	5	−1.2	−2.1	not assigned.no ontology	STAM-binding protein,
cBR_36	1	1	2	7	−2.2	−1.1	not assigned.unknown	expressed protein
cBR_37	1	1	2	3	3.1	−2.9	not assigned.no ontology	abhydrolase domain-containing protein 5,
cBR_38	1	1	2	8	−1.8	−5.5	stress.abiotic.heat	heat shock 70 kDa protein 1,
cBR_39	1	1	2	4	1.1	1.6	cell.organisation	myosin Ie,
cBR_40	1	1	2	1	5.7	1.9	secondary metabolism.flavonoids.flavonols	flavonol synthase/flavanone 3-hydroxylase,
cBR_41	1	1	2	4	1.3	5.6	lipid metabolism.FA synthesis and FA elongation.long chain fatty acid CoA ligase	acyl-CoA synthetase,
cBR_42	1	1	2	5	−1.2	−2.6	protein.degradation.ubiquitin.E3.HECT	thyroid receptor-interacting protein 12,
cBR_43	1	1	2	8	−1.4	−2.3	development.storage proteins	protein COQ10 A, mitochondrial precursor,
cBR_44	1	1	2	2	13.3	−1.1	nucleotide metabolism.synthesis.purine.amidophosphoribosyltransferase	amidophosphoribosyltransferase, chloroplast precursor,
cBR_45	1	1	2	4	−1.1	7.2	transport.p- and v-ATPases.H+-transporting two-sector ATPase	vacuolar ATP synthase subunit E,
cBR_46	1	1	2	8	−2.1	−3.8	protein.degradation.AAA type	ATP binding protein,
cBR_47	1	1	2	8	−1.7	−1.5	protein.degradation.ubiquitin.E3.RING	RING zinc finger protein,
cBR_48	1	1	2	8	−2.4	−2.6	not assigned.unknown	expressed protein
cBR_49	1	1	2	3	2.5	−2.3	not assigned.unknown	NO_MATCH
cBR_50	1	1	2	8	−2.5	−1.7	RNA.regulation of transcription.HDA	histone deacetylase 11,
cBR_51	1	1	2	1	2.0	2.3	stress.abiotic.drought/salt	ankyrin protein kinase-like,
cBR_52	1	1	2	8	−1.5	−1.5	not assigned.no ontology	retrotransposon protein, putative, Ty3-gypsy subclass
cBR_53	1	1	2	1	2.8	2.1	cell.organisation	actin-1,
cBR_54	1	1	2	4	−1.0	1.9	not assigned.unknown	expressed protein
cBR_55	1	1	2	3	2.9	−2.7	protein.degradation.ubiquitin.E3.SCF.FBOX	adagio protein 1,
cBR_56	1	1	2	4	1.3	8.3	hormone metabolism.jasmonate.synthesis-degradation.12-Oxo-PDA-reductase	12-oxophytodienoate reductase 2,
cBR_57	1	1	2	3	1.8	−2.4	misc.glutathione S transferases	glutathione S-transferase GSTU6,
cBR_58	1	1	2	4	1.0	1.9	protein.postranslational modification.kinase.receptor like cytoplasmatic kinase VII	ATP binding protein,
cBR_59	1	1	2	1	3.6	1.4	signalling.G-proteins	prenylated Rab receptor 2,
cBR_60	1	1	2	4	−1.0	9.7	not assigned.no ontology	plant-specific FAD-dependent oxidoreductase family protein
cBR_61	1	1	2	8	−2.0	−2.0	not assigned.unknown	expressed protein
cBR_62	1	1	2	1	1.4	2.5	not assigned.unknown	expressed protein
cBR_63	1	1	2	8	−2.2	−2.1	not assigned.no ontology	transferase, transferring glycosyl groups,
cBR_64	1	1	2	4	−1.1	2.4	not assigned.no ontology.armadillo/beta-catenin repeat family protein	armadillo/beta-catenin-like repeat family protein, expressed
cBR_65	1	1	2	1	1.7	3.4	misc.nitrilases, *nitrile lyases, berberine bridge enzymes, reticuline oxidases, troponine reductases	amidase,
cBR_66	1	1	2	6	−1.9	1.4	not assigned.no ontology.pentatricopeptide (PPR) repeat-containing protein	EMB2748,
cBR_67	1	1	2	4	−1.3	3.6	development.storage proteins	legumin-like protein,
cBR_68	1	1	2	2	6.7	1.2	misc.peroxidases	peroxidase 24 precursor,
cBR_69	1	1	2	2	7.3	−1.3	misc.peroxidases	peroxidase 65 precursor,
cBR_70	1	1	2	7	−2.5	−1.1	not assigned.no ontology	regulator of ribonuclease activity A,
cBR_71	1	1	2	8	−1.4	−1.4	signalling.14-3-3 proteins	14-3-3-like protein,
cBR_72	1	1	2	1	7.7	1.4	misc.UDP glucosyl and glucoronyl transferases	glycosyltransferase 5,
cBR_73	1	1	2	4	−1.0	7.2	RNA.regulation of transcription.DNA methyltransferases	DNA cytosine methyltransferase MET2a,
cBR_74	1	1	2	5	−1.2	−1.5	signalling.G-proteins	ras-related protein Rab-2-A,
cBR_75	1	1	2	5	−1.1	−1.9	stress.abiotic.heat	chaperone protein dnaJ 10,
cBR_76	1	1	2	7	−1.6	1.0	not assigned.unknown	SEC6,
cBR_77	1	1	2	3	2.6	−1.6	signalling.calcium	calcium-dependent protein kinase, isoform AK1,
cBR_78	1	1	2	7	−2.4	−1.4	not assigned.unknown	expressed protein
cBR_79	1	1	2	3	2.3	−2.3	signalling.G-proteins	TBC domain containing protein, expressed
cBR_80	1	1	2	2	2.3	1.3	protein.postranslational modification	transposon protein, putative, unclassified, expressed
cBR_81	1	1	2	4	−1.2	3.0	protein.synthesis.misc ribosomal protein	60 ribosomal protein L14,
cBR_82	1	1	2	8	−2.0	−3.8	protein.degradation.cysteine protease	OTU-like cysteine protease family protein, expressed
cBR_83	1	1	2	8	−1.8	−1.6	not assigned.unknown	protein phosphatase inhibitor containing protein, expressed
cBR_84	1	1	2	1	2.4	4.8	not assigned.unknown	NO_MATCH
cBR_85	1	1	2	8	−1.7	−3.8	misc.cytochrome P450	cytochrome P450 72A1,
cBR_86	1	1	2	1	1.4	1.7	not assigned.unknown	expressed protein
cBR_87	1	1	2	2	5.1	−1.2	lipid metabolism.FA synthesis and FA elongation.beta ketoacyl CoA synthase	3-ketoacyl-CoA synthase,
cBR_88	1	1	2	5	−1.3	−1.5	development.unspecified	tRNA 2phosphotransferase,
cBR_89	1	1	2	7	−1.5	−1.3	misc.acid and other phosphatases	lipid phosphate phosphatase 3, chloroplast precursor,
cBR_90	1	1	2	4	1.1	15.1	protein.synthesis.misc ribosomal protein	60S acidic ribosomal protein P2A,
cBR_91	1	1	2	4	1.1	4.9	cell wall.cell wall proteins.AGPs	fasciclin-like arabinogalactan protein 10 precursor,
cBR_92	1	1	2	3	12.1	−2.7	not assigned.unknown	expressed protein
cBR_93	1	1	2	5	−1.3	−1.5	protein.postranslational modification	calcium-dependent protein kinase,
cBR_94	1	1	2	4	1.1	13.6	misc.peroxidases	peroxidase 17 precursor,
cBR_95	1	1	2	5	−1.1	−3.8	not assigned.unknown	NO_MATCH
cBR_96	1	1	2	3	5.1	−2.7	protein.postranslational modification	EDR1,
cBR_97	1	1	2	2	4.0	−1.2	not assigned.no ontology	mitochondrial prohibitin complex protein 2,
cBR_98	1	1	2	5	−1.1	−1.8	not assigned.unknown	expressed protein
cBR_99	1	1	2	8	−1.7	−1.8	cell.organisation	ATPP2-A13,
cBR_100	1	1	2	4	−1.0	3.6	transport.peptides and oligopeptides	peptide transporter PTR2,
cBR_101	1	1	2	4	−1.2	3.5	PS.lightreaction.ATP synthase	ATP synthase beta chain, mitochondrial precursor,
cBR_102	1	1	2	7	−1.4	−1.0	not assigned.no ontology	WD-repeat protein pop3,
cBR_103	1	1	2	8	−2.6	−2.3	not assigned.unknown	steroid nuclear receptor, ligand-binding,
cBR_104	1	1	2	4	−1.1	1.9	lipid metabolism.glyceral metabolism.Glycerol-3-phosphate dehydrogenase (NAD+)	glycerol-3-phosphate dehydrogenase,
cBR_105	1	1	2	8	−1.4	−2.3	not assigned.no ontology	protein YIF1A,
cBR_106	1	1	2	1	1.6	1.9	not assigned.unknown	acid phosphatase/vanadium-dependent haloperoxidase related,
cBR_107	1	1	2	5	1.2	−2.4	protein.degradation.ubiquitin.E3.SCF.FBOX	F-box/LRR-repeat MAX2,
cBR_108	1	1	2	1	5.3	1.9	nucleotide metabolism.phosphotransfer and pyrophosphatases.misc	ectonucleotide pyrophosphatase/phosphodiesterase 1,
cBR_109	1	1	2	7	−2.0	−1.3	transport.metal	metal tolerance protein C3,
cBR_110	1	1	2	8	−2.0	−1.5	protein.targeting.nucleus	trans-2-enoyl-CoA reductase, mitochondrial precursor,
cBR_111	1	1	2	4	−1.2	4.1	protein.synthesis.misc ribosomal protein	60S ribosomal protein L44,
cBR_112	1	1	2	7	−2.5	−1.2	protein.degradation.ubiquitin.E3.SCF.FBOX	F-box domain containing protein, expressed
cBR_113	1	1	2	5	1.2	−2.5	not assigned.no ontology	EMB1374,
cBR_114	1	1	2	2	1.8	1.1	N-metabolism.nitrate metabolism.NR	cytochrome b5,
cBR_115	1	1	2	6	−1.9	2.0	not assigned.unknown	expressed protein
cBR_116	1	1	2	4	1.1	2.2	amino acid metabolism.synthesis.aromatic aa.tryptophan.tryptophan synthase	indole-3-glycerol phosphate lyase, chloroplast precursor,
cBR_117	1	1	2	1	3.3	9.1	misc.gluco-, galacto- and mannosidases	beta-glucosidase homolog precursor,
cBR_118	1	1	2	4	−1.1	3.4	protein.synthesis.misc ribosomal protein	60S ribosomal protein L33-B,
cBR_119	1	1	2	4	1.0	6.6	secondary metabolism.isoprenoids.mevalonate pathway.HMG-CoA synthase	hydroxymethylglutaryl-CoA synthase,
cBR_120	1	1	2	8	−1.8	−5.5	misc.short chain dehydrogenase/reductase (SDR)	general stress protein 39,
cBR_121	1	1	2	1	2.0	2.0	not assigned.no ontology	monoglyceride lipase,
cBR_122	1	1	2	4	−1.0	9.1	not assigned.unknown	expressed protein
cBR_123	1	1	2	8	−1.8	−7.0	Biodegradation of Xenobiotics.lactoylglutathione lyase	lactoylglutathione lyase,
cBR_124	1	1	2	8	−2.0	−1.4	cell.cycle.peptidylprolyl isomerase	peptidyl-prolyl isomerase,
cBR_125	1	1	2	5	−1.3	−1.6	not assigned.unknown	NO_MATCH
cBR_126	1	1	2	3	2.6	−2.5	minor CHO metabolism.trehalose.TPP	expressed protein
cBR_127	1	1	2	5	1.0	−2.4	metal handling	selenium-binding protein,
cBR_128	1	1	2	1	1.8	2.2	protein.degradation.ubiquitin.proteasom	proteasome subunit alpha type 7,
cBR_129	1	1	2	1	32.5	3.5	cell wall.modification	beta-expansin 1a precursor,
cBR_130	1	1	2	6	−1.4	41.0	stress.abiotic.unspecified	oxalate oxidase 2 precursor,
cBR_131	1	1	2	1	4.3	3.2	not assigned.unknown	expressed protein
cBR_132	1	1	2	3	2.1	−2.0	misc.cytochrome P450	cytochrome P450 72A1,
cBR_133	1	1	2	6	−1.9	1.5	not assigned.unknown	NO_MATCH
cBR_134	1	1	2	1	1.4	7.2	C1-metabolism	methylenetetrahydrofolate reductase,
cBR_135	1	1	2	1	1.9	3.7	not assigned.no ontology	seed maturation protein,
cBR_136	1	1	2	5	−1.2	−2.1	stress.abiotic.cold	USP family protein,
cBR_137	1	1	2	8	−1.4	−3.4	development.unspecified	caleosin 2,
cBR_138	1	1	2	4	1.1	2.2	protein.degradation.ubiquitin.proteasom	26S protease regulatory subunit S10B,
cBR_139	1	1	2	1	1.4	2.1	not assigned.no ontology	translocon-associated protein beta containing protein, expressed
cBR_140	1	1	2	5	−1.2	−2.2	not assigned.unknown	holocarboxylase synthetase,
cBR_141	1	1	2	2	2.1	1.3	signalling.G-proteins	GTP-binding protein SAR1A,
cBR_142	1	1	2	6	−1.7	4.0	not assigned.no ontology	wound/stress protein,
cBR_143	1	1	2	8	−1.5	−4.6	not assigned.no ontology	nifU-like N-terminal domain containing protein, mitochondrial precursor,
cBR_144	1	1	2	8	−2.2	−4.7	minor CHO metabolism.others	aldose reductase,
cBR_145	1	1	2	4	1.1	33.0	major CHO metabolism.degradation.starch.starch cleavage	alpha-amylase precursor,
cBR_146	1	1	2	1	1.5	5.7	secondary metabolism.phenylpropanoids.lignin biosynthesis.CCoAOMT	caffeoyl-CoA O-methyltransferase 1,
cBR_147	1	1	2	1	2.6	3.6	cell.organisation	tubulin alpha-1 chain,
cBR_148	1	1	2	3	4.1	−2.2	glycolysis.pyrophosphate-fructose-6-P phosphotransferase	pyrophosphate–fructose 6-phosphate 1-phosphotransferase alpha subunit,
cBR_149	1	1	2	1	2.8	1.6	transport.metabolite transporters at the envelope membrane	plastidic phosphate translocator-like protein1,
cBR_150	1	1	2	8	−1.5	−2.1	RNA.regulation of transcription.unclassified	DNL zinc finger family protein, expressed
cBR_151	1	1	2	1	2.8	1.5	misc.misc2	glycosyltransferase 48 kDasubunit precursor,
cBR_152	1	1	2	4	−1.1	5.5	misc.acid and other phosphatases	tartrate-resistant acid phosphatase type 5 precursor,
cBR_153	1	1	2	4	1.2	4.0	protein.aa activation.bifunctional aminoacyl-tRNA synthetase	bifunctional aminoacyl-tRNA synthetase,
cBR_154	1	1	2	4	−1.0	2.9	protein.postranslational modification	serine/threonine protein phosphatase 2A
cBR_155	1	1	2	5	−1.3	−2.3	not assigned.no ontology	BCL-2 binding anthanogene-1,
cBR_156	1	1	2	1	1.8	2.5	TCA/org. transformation.TCA.pyruvate DH.E1	pyruvate dehydrogenase E1 component alpha subunit,
cBR_157	1	1	2	8	−1.8	−1.8	not assigned.no ontology	hypersensitive-induced response protein,
cBR_158	1	1	2	4	1.1	4.7	cell wall.precursor synthesis.phosphomannomutase	phosphomannomutase,
cBR_159	1	1	2	4	−1.2	2.1	signalling.G-proteins	ras-related protein Rab11A,
cBR_160	1	1	2	1	1.6	1.7	signalling.G-proteins	ras-related protein ARA-3,
cBR_161	1	1	2	1	4.9	1.6	cell wall.degradation.cellulases and beta -1,4-glucanases	periplasmic beta-glucosidase precursor,
cBR_162	1	1	2	4	−1.0	3.4	not assigned.unknown	NO_MATCH
cBR_163	1	1	2	1	2.3	13.7	metal handling.binding, chelation and storage	nicotianamine synthase 3,
cBR_164	1	1	2	1	1.8	25.1	DNA.synthesis/chromatin structure	DNA replication licensing factor mcm4,
cBR_165	1	1	2	4	1.1	4.9	lipid metabolism.exotics (steroids, squalene etc)	minor allergen Alt a 7,
cBR_166	1	1	2	7	−1.6	−1.2	not assigned.no ontology	IWS1 C-terminus family protein, expressed
cBR_167	1	1	2	1	1.9	10.3	protein.degradation.cysteine protease	vignain precursor,
cBR_168	1	1	2	4	1.2	57.3	secondary metabolism.phenylpropanoids.lignin biosynthesis.COMT	quercetin 3-O-methyltransferase 1,
cBR_169	1	1	2	1	2.9	11.9	development.unspecified	pollen-specific protein SF3,
cBR_170	1	1	2	1	2.7	1.5	not assigned.unknown	expressed protein
cBR_171	1	1	2	8	−1.8	−1.8	Biodegradation of Xenobiotics.lactoylglutathione lyase	glyoxalase family protein superfamily,
cBR_172	1	1	2	2	4.2	1.3	lipid metabolism.FA synthesis and FA elongation.pyruvate DH	pyruvate dehydrogenase E1 component subunit beta,
cBR_173	1	1	2	5	−1.1	−1.8	misc.misc2	epoxide hydrolase 2,
cBR_174	1	1	2	6	−1.6	2.8	amino acid metabolism.synthesis.branched chain group.leucine specific.3-isopropylmalate dehydrogenase	3-isopropylmalate dehydrogenase 2, chloroplast precursor,
cBR_175	1	1	2	3	7.8	−1.7	protein.postranslational modification.kinase.receptor like cytoplasmatic kinase VII	protein kinase,
cBR_176	1	1	2	3	1.7	−3.0	secondary metabolism.isoprenoids.tocopherol biosynthesis.hydroxyphenylpyruvate dioxygenase	4-hydroxyphenylpyruvate dioxygenase
cBR_177	1	1	2	6	−1.5	1.7	not assigned.unknown	expressed protein
cBR_178	1	1	2	8	−2.1	−2.4	transport.NDP-sugars at the ER	solute carrier family 35 member B3,
cBR_179	1	1	2	6	−1.4	1.8	not assigned.unknown	NO_MATCH
cBR_180	1	1	2	8	−2.6	−1.9	signalling.calcium	calmodulin-related protein 2, touch-induced,
cBR_181	1	1	2	1	2.0	2.5	not assigned.no ontology.C2 domain-containing protein	elicitor-responsive protein 3,
cBR_182	1	1	2	4	−1.1	5.2	lipid metabolism.glyceral metabolism.glycerol kinase	glycerol kinase,
cBR_183	1	1	2	1	2.8	13.3	protein.degradation.serine protease	serine carboxypeptidase 3 precursor,
cBR_184	1	1	2	4	−1.2	2.6	not assigned.unknown	expressed protein
cBR_185	1	1	2	3	1.7	−5.9	not assigned.no ontology	WD-repeat protein-like,
cBR_186	1	1	2	8	−3.1	−2.0	not assigned.no ontology.C2 domain-containing protein	calcium lipid binding protein-like,
cBR_187	1	1	2	3	6.7	−3.8	protein.degradation.AAA type	cell Division Protein AAA ATPase family,
cBR_188	1	1	2	3	17.3	−11.1	not assigned.unknown	nematode-resistance protein,
cBR_189	1	1	2	5	−1.3	−2.0	not assigned.unknown	seed maturation protein PM23,
cBR_190	1	1	2	4	1.2	4.3	transporter.sugars	major myo-inositol transporter iolT,
cBR_191	1	1	2	5	−1.0	−2.8	RNA.regulation of transcription.AP2/EREBP, APETALA2/Ethylene-responsive element binding protein family	AP2/EREBP, APETALA2/Ethylene-responsive element binding protein family
cBR_192	1	1	2	4	1.0	5.0	amino acid metabolism.synthesis.serine-glycine-cysteine group.serine.phosphoserine phosphatase	phosphoserine phosphatase, chloroplast precursor,
cBR_193	1	1	2	2	3.1	1.3	signalling.calcium	calmodulin,
cBR_194	1	1	2	1	1.4	6.6	N-metabolism.N-degradation.glutamate dehydrogenase	glutamate dehydrogenase,
cBR_195	1	1	2	1	2.9	4.3	not assigned.unknown	GPI-anchored protein At5g19240 precursor,
cBR_196	1	1	2	6	−1.4	2.3	not assigned.unknown	brain protein 44-like protein,
cBR_197	1	1	2	4	1.0	3.9	not assigned.unknown	expressed protein
cBR_198	1	1	2	5	−1.1	−1.9	cell.vesicle transport	syntaxin 23,
cBR_199	1	1	2	1	6.4	8.3	cell wall.degradation.mannan-xylose-arabinose-fucose	beta-D-xylosidase,
cBR_200	1	1	2	1	1.6	1.8	not assigned.unknown	NO_MATCH
cBR_201	1	1	2	1	8.6	3.8	misc.plastocyanin-like	blue copper protein precursor,
cBR_202	1	1	2	5	−1.3	−2.9	protein.postranslational modification	peptide methionine sulfoxide reductase,
cBR_203	1	1	2	4	1.2	3.6	protein.synthesis.misc ribosomal protein	40S ribosomal protein S3a,
cBR_204	1	1	2	1	16.9	2.4	cell wall.modification	beta-expansin 1a precursor,
cBR_205	1	1	2	2	6.4	−1.2	misc.glutathione S transferases	glutathione S-transferase GSTU6,
cBR_206	1	1	2	4	−1.1	2.1	RNA.processing.ribonucleases	ribonuclease 2 precursor,
cBR_207	1	1	2	5	−1.2	−1.4	RNA.regulation of transcription.Alfin-like	PHD finger protein,
cBR_208	1	1	2	1	2.0	3.5	lipid metabolism.lipid degradation.lysophospholipases.phospholipase A2	phospholipase A2,
cBR_209	1	1	2	5	−1.3	−1.6	protein.degradation.ubiquitin.E3.RING	protein binding protein,
cBR_210	1	1	2	2	2.0	1.3	not assigned.unknown	expressed protein
cBR_211	1	1	2	1	2.6	6.3	lipid metabolism.FA desaturation.omega 3 desaturase	omega-3 fatty acid desaturase
cBR_212	1	1	2	5	−1.2	−3.1	signalling.G-proteins	GTP binding protein,
cBR_213	1	1	2	5	−1.0	−3.8	protein.postranslational modification	protein phosphatase 2C isoform epsilon,
cBR_214	1	1	2	4	1.1	2.2	TCA/org. transformation.other organic acid transformaitons.atp-citrate lyase	ATP-citrate synthase,
cBR_215	1	1	2	8	−2.3	−2.5	Biodegradation of Xenobiotics.lactoylglutathione lyase	lactoylglutathione lyase,
cBR_216	1	1	2	5	−1.2	−2.1	RNA.transcription	transcription initiation factor TFIID subunit 10,
cBR_217	1	1	2	1	11.8	8.4	cell wall.cell wall proteins.AGPs	fasciclin-like arabinogalactan protein 7 precursor,
cBR_218	1	1	2	5	−1.3	−2.3	not assigned.unknown	expressed protein
cBR_219	1	1	2	3	1.7	−1.8	protein.degradation.AAA type	ATP binding protein,
cBR_220	1	1	2	4	1.3	2.5	cell.vesicle transport	AP-2 complex subunit sigma-1,
cBR_221	1	1	2	1	2.3	3.9	RNA.transcription	DNA-directed RNA polymerases II 24 kDa polypeptide,
cBR_222	1	1	2	8	−1.6	−1.7	not assigned.no ontology	deoxyribonuclease ycfH,
cBR_223	1	1	2	4	1.3	2.0	protein.synthesis.ribosomal protein.prokaryotic	50S ribosomal protein L11, chloroplast precursor,
cBR_224	1	1	2	4	1.3	2.7	RNA.transcription	DNA-directed RNA polymerases I and III 14 kDa polypeptide,
cBR_225	1	1	2	8	−1.8	−5.2	major CHO metabolism.degradation.starch.starch cleavage	beta-amylase,
cBR_226	1	1	2	8	−2.2	−1.9	not assigned.unknown	expressed protein
cBR_227	1	1	2	6	−1.6	2.7	protein.synthesis.ribosomal protein.unknown	structural constituent of ribosome,
cBR_228	1	1	2	4	−1.1	3.3	cell wall.degradation.mannan-xylose-arabinose-fucose	beta-D-xylosidase,
cBR_229	1	1	2	4	−1.2	4.1	not assigned.unknown	NO_MATCH
cBR_230	1	1	2	8	−1.4	−2.0	amino acid metabolism.synthesis.serine-glycine-cysteine group.cysteine.SAT	serine acetyltransferase 2,
cBR_231	1	1	2	8	−1.6	−1.4	not assigned.no ontology	hydrolase, NUDIX family protein, expressed
cBR_232	1	1	2	5	−1.0	−1.8	not assigned.unknown	fos intronic gene CG7615-PA,
cBR_233	1	1	2	8	−1.4	−2.1	cell.cycle	cyclin-T1,
cBR_234	1	1	2	1	1.5	3.6	N-metabolism.ammonia metabolism.unspecified	haloacid dehalogenase-like hydrolase domain-containing protein 1A,
cBR_235	1	1	2	8	−1.7	−2.8	not assigned.unknown	NO_MATCH
cBR_236	1	1	2	6	−1.4	2.7	nucleotide metabolism.synthesis.purine.GMP synthetase	GMP synthase,
cBR_237	1	1	2	4	1.3	3.0	not assigned.unknown	NO_MATCH
cBR_238	1	1	2	2	2.6	−1.0	protein.postranslational modification	CENP-E like kinetochore protein,
cBR_239	1	1	2	4	−1.3	3.7	protein.targeting.mitochondria	mitochondrial import inner membrane translocase subunit tim22,
cBR_240	1	1	2	1	1.5	5.3	signalling.G-proteins	ADP-ribosylation factor-like protein 8B,
cBR_241	1	1	2	1	3.4	2.1	signalling.calcium	calcium-transporting ATPase 4, plasma membrane-type,
cBR_242	1	1	2	2	20.3	1.2	N-metabolism.nitrate metabolism.NR	desaturase/cytochrome b5 protein,
cBR_243	1	1	2	3	3.2	−2.1	not assigned.unknown	expressed protein
cBR_244	1	1	2	5	−1.3	−1.9	misc.glutathione S transferases	glutathione S-transferase GSTU6,
cBR_245	1	1	2	8	−2.0	−1.7	protein.degradation.ubiquitin.E3.SCF.FBOX	F-box domain containing protein, expressed
cBR_246	1	1	2	8	−2.4	−1.8	signalling.G-proteins	ras-related protein Rab-18,
cBR_247	1	1	2	3	4.6	−2.4	cell wall.pectin*esterases.PME	pectinesterase-1 precursor,
cBR_248	1	1	2	7	−4.5	−1.0	transport.metal	zinc transporter 10 precursor
cBR_249	1	1	2	8	−2.1	−2.7	not assigned.unknown	expressed protein
cBR_250	1	1	2	1	2.8	1.5	protein.synthesis.ribosomal protein.prokaryotic	succinate dehydrogenase iron-sulfur protein,mitochondrial precursor,
cBR_251	1	1	2	4	1.1	4.7	DNA.synthesis/chromatin structure.histone	histone H2A,
cBR_252	1	1	2	4	−1.0	6.1	lipid metabolism.exotics (steroids, squalene etc)	flavonol 4-sulfotransferase,
cBR_253	1	1	2	7	−1.9	−1.3	protein.degradation.serine protease	serine carboxypeptidase 1 precursor,
cBR_254	1	1	2	8	−1.5	−2.4	cell.vesicle transport	syntaxin 132,
cBR_255	1	1	2	1	3.1	8.0	misc.oxidases - copper, flavone etc.	L-ascorbate oxidase homolog precursor,
cBR_256	1	1	2	1	2.3	4.7	transport.unspecified anions	UDP-glucose 6-dehydrogenase,
cBR_257	1	1	2	1	2.2	3.2	transport.metabolite transporters at the mitochondrial membrane	mitochondrial 2-oxoglutarate/malate carrier protein,
cBR_258	1	1	2	8	−1.7	−2.7	not assigned.no ontology	regulatory subunit,
cBR_259	1	1	2	5	−1.1	−2.0	RNA.regulation of transcription.unclassified	PAPA-1-like conserved region family protein, expressed
cBR_260	1	1	2	8	−1.9	−3.0	misc.misc2	oxidoreductase,
cBR_261	1	1	2	6	−1.4	4.3	misc.dynamin	ATP binding protein,
cBR_262	1	1	2	8	−1.4	−2.9	not assigned.no ontology	ADP-ribosylation factor GTPase-activating protein 3,
cBR_M1	1	2	3	1	9.1	1.6	cell wall.cellulose synthesis.cellulose synthase	CESA1 - cellulose synthase, expressed
cBR_M2	17	3	20	4	1.2	22.4	protein.degradation.ubiquitin.E3.RING	U-box domain containing protein, expressed
cBR_M3	1	2	3	6	−1.4	4.6	protein.synthesis.misc ribosomal protein	60S ribosomal protein L24,
cBR_M4	2	1	3	4	−1.0	2.9	misc.GDSL-motif lipase	esterase precursor,
cBR_M5	1	2	3	2	4.0	−1.1	PS.lightreaction.other electron carrier (ox/red).ferredoxin	ferredoxin-3, chloroplast precursor,
cBR_M6	1	2	3	8	−1.4	−1.8	stress.biotic	tobamovirus multiplication 3,
cBR_M7	1	2	3	3	11.4	−4.7	protein.degradation.AAA type	ATPase 2,
cBR_M8	1	3	4	7	−2.2	1.0	cell.organisation	myosin XI,
cBR_M9	1	2	3	4	1.2	1.8	signalling.G-proteins	ras-related protein ARA-3,
cBR_M10	1	3	4	1	2.6	2.1	cell.organisation	tubulin beta-5 chain,
cBR_M11	2	2	4	4	−1.1	6.6	not assigned.unknown	lysine decarboxylase-like protein,
cBR_M13	3	2	5	8	−2.0	−3.5	transport.misc	ABA induced plasma membrane protein PM 19,
cBR_M14	1	2	3	8	−4.5	−7.3	minor CHO metabolism.raffinose family.galactinol synthases.putative	galactinol synthase 3,
cBR_M15	1	6	7	1	1.9	2.1	signalling.receptor kinases.misc	receptor-like protein kinase 5 precursor,
cBR_M16	1	3	4	1	1.8	2.2	protein.degradation.ubiquitin.E2	ubiquitin-conjugating enzyme E2–17 kDa,
cBR_M17	3	5	8	1	13.0	1.4	signalling.G-proteins	ras-related protein ARA-4,
cBR_M18	3	4	7	1	15.0	6.5	misc.peroxidases	peroxidase 52 precursor,
cBR_M19	2	3	5	5	−1.2	−2.8	protein.postranslational modification	serine/threonine-protein kinase SAPK6,
cBR_M20	1	2	3	1	2.6	2.0	Co-factor and vitamine metabolism	pyridoxin biosynthesis protein ER1,
cBR_M22	1	2	3	4	−1.0	1.5	cell.organisation	actin-7,
cBR_M23	8	2	10	6	−1.5	28.8	DNA.synthesis/chromatin structure.histone	histone H4,
cBR_M24	2	1	3	4	1.2	40.1	DNA.synthesis/chromatin structure.histone	histone H2A,
cBR_M25	8	1	9	4	1.2	39.4	DNA.synthesis/chromatin structure.histone	histone H2A,
cBR_M26	3	3	6	1	1.7	17.1	DNA.synthesis/chromatin structure	DNA replication licensing factor Mcm2,
cBR_M27	1	2	3	5	−1.3	−3.4	not assigned.no ontology	transmembrane BAX inhibitor motif-containing protein 4,
cBR_M28	1	2	3	5	−1.1	−2.3	protein.degradation.ubiquitin.E3.RING	protein binding protein,
cBR_M29	1	2	3	6	−2.0	1.4	not assigned.no ontology	ubiquitin carboxyl-terminal hydrolase 21,
cBR_M31	2	3	5	8	−1.7	−5.0	misc.short chain dehydrogenase/reductase (SDR)	expressed protein
cBR_M32	2	1	3	1	1.4	2.1	stress.abiotic	CAB2,
cBR_M33	1	2	3	8	−2.8	−4.6	not assigned.unknown	expressed protein
cBR_M34	1	5	6	3	1.6	−1.9	protein.postranslational modification.kinase.receptor like cytoplasmatic kinase VII	serine/threonine-protein kinase NAK,
cBR_M35	2	1	3	8	−1.7	−2.4	not assigned.no ontology	diacylglycerol O-acyltransferase 1,
cBR_M36	1	2	3	8	−2.5	−2.2	transporter.membrane system unknown	glycerol 3-phosphate permease,
cBR_M37	1	3	4	1	2.8	1.7	signalling.14-3-3 proteins	14-3-3-like protein A,
cBR_M39	1	2	3	1	1.5	53.3	DNA.synthesis/chromatin structure.histone	histone H2A variant 2,
cBR_M40	1	2	3	3	13.9	−1.7	cell wall.modification	alpha-expansin 6 precursor,
cBR_M41	1	3	4	4	1.3	3.3	signalling.G-proteins	rac-like GTP-binding protein 6,
cBR_M42	1	2	3	2	2.9	−1.2	protein.degradation.ubiquitin.E2	ubiquitin-conjugating enzyme E2 I,
cBR_M43	2	1	3	3	10.0	−5.2	cell wall.modification	xyloglucan endotransglucosylase/hydrolase protein 23 precursor,
cBR_M46	2	1	3	2	3.2	−1.2	not assigned.unknown	protein GPR108 precursor,
cBR_M47	1	2	3	3	24.2	−3.0	not assigned.unknown	expressed protein
cBR_M48	2	2	4	1	1.8	3.5	signalling.receptor kinases.leucine rich repeat XI	receptor-like protein kinase precursor,
cBR_M49	1	3	4	1	1.5	3.7	misc.cytochrome P450	cytochrome P450 71D8,
cBR_M50	2	1	3	4	1.3	7.2	cell wall.cellulose synthesis	CSLC3 - cellulose synthase-like family C, expressed
cBR_M51	2	1	3	4	1.1	2.9	misc.O-methyl transferases	adenylate kinase B,
cBR_M52	1	2	3	4	−1.2	6.3	lipid metabolism.lipid degradation.lysophospholipases.glycerophosphodiester phosphodiesterase	glycerophosphoryl diester phosphodiesterase precursor,
cBR_M53	2	2	4	4	1.2	4.2	protein.synthesis.misc ribosomal protein	60S ribosomal protein L10a-1,
cBR_M54	1	2	3	4	−1.1	9.6	stress.abiotic.unspecified	rhicadhesin receptor precursor,
cBR_M55	1	1	2	4	−1.3	7.0	misc.UDP glucosyl and glucoronyl transferases	cis-zeatin O-glucosyltransferase,
cBR_M56	1	2	3	8	−1.8	−1.6	development.unspecified	ubiquitin ligase SINAT3,
cBR_M57	2	1	3	8	−2.1	−1.8	not assigned.no ontology	membrane protein,
cBR_M58	1	2	3	4	−1.0	31.0	cell.cycle	cyclin IIIZm,
cBR_M59	1	2	3	5	−1.2	−2.3	stress.abiotic.heat	dnaJ homolog subfamily B member 5,
cBR_M60	1	2	3	1	1.5	30.1	not assigned.unknown	NO_MATCH

### Diverse Gene Expression Patterns Were Preserved in Barley and Rice Germination

There are eight possible expression patterns based on up or down-regulations of a gene in early and late germination phases. All of the possible expression patterns were observed for the cBRs, and were preserved in both rice and barely since their divergence (Table 3 and 4). Table 4 summarized the cBRs in the eight expression patterns. A total of 71 cBRs showed up-regulated expression patterns in both early and late germination phases, and made up the largest group of cBRs (Group 1). Many cBRs in the Group 1 encoded the proteins related to cell wall metabolism, cell organization, chromatin structure, protein degradation, and signaling G-proteins.

**Table 4:** Summary of cBR Expression Patterns.

Group	early phase	late phase	No. of cBRs
**1**	Up	Up	71
**2**	Up	No	18
**3**	Up	Down	28
**4**	No	Up	69
**5**	No	Down	36
**6**	Down	Up	17
**7**	Down	No	13
**8**	Down	Down	62

Note: The cut-off value for the Up, Down and No change of cBR expression in early and late germination phase is 1.4-fold change.

Interestingly, Group 3 had 28 cBRs that were transiently up-regulated in the early germination phase. Expression levels of most cBRs in Group 3 at the end of germination were down-regulated to levels at the dry seed stage. Preserving transient up-regulation in early germination followed by down-regulation in late germination in both barley and rice indicated that those genes likely participated in biological processes specific to early germination. Many cBRs in Group 3 encoded proteins involved in cell wall modification, protein degradation, protein modification, and signaling transduction. Cell wall modification is required to weaken cell walls during early germination to permit radicle protrusion and to provide access to stored metabolites in the endosperm [23]. Also in Group 3 were proteins such as F-box proteins, receptor-like kinases, G-proteins and calcium-dependent protein kinases, which play important roles in a variety of signaling transduction pathways. Those signaling components likely played roles in transducing a variety of signals in the early germination phase to initiate the biological pathways required in seed germination. Sixty-two cBRs in Group 8 were down-regulated in both early and late stages. They encoded proteins with a wide range of biological functions. Those cBRs highly accumulated in dry mature grains and their accumulation gradually decreased over the course of seed germination. This raises the possibility that these cBRs encoded proteins involved in seed development and maturation. The highly accumulated transcripts were degraded over the course of seed germination.

### The cBRs Encoded Proteins in Diverse Biological Pathways

**Figure 2:**
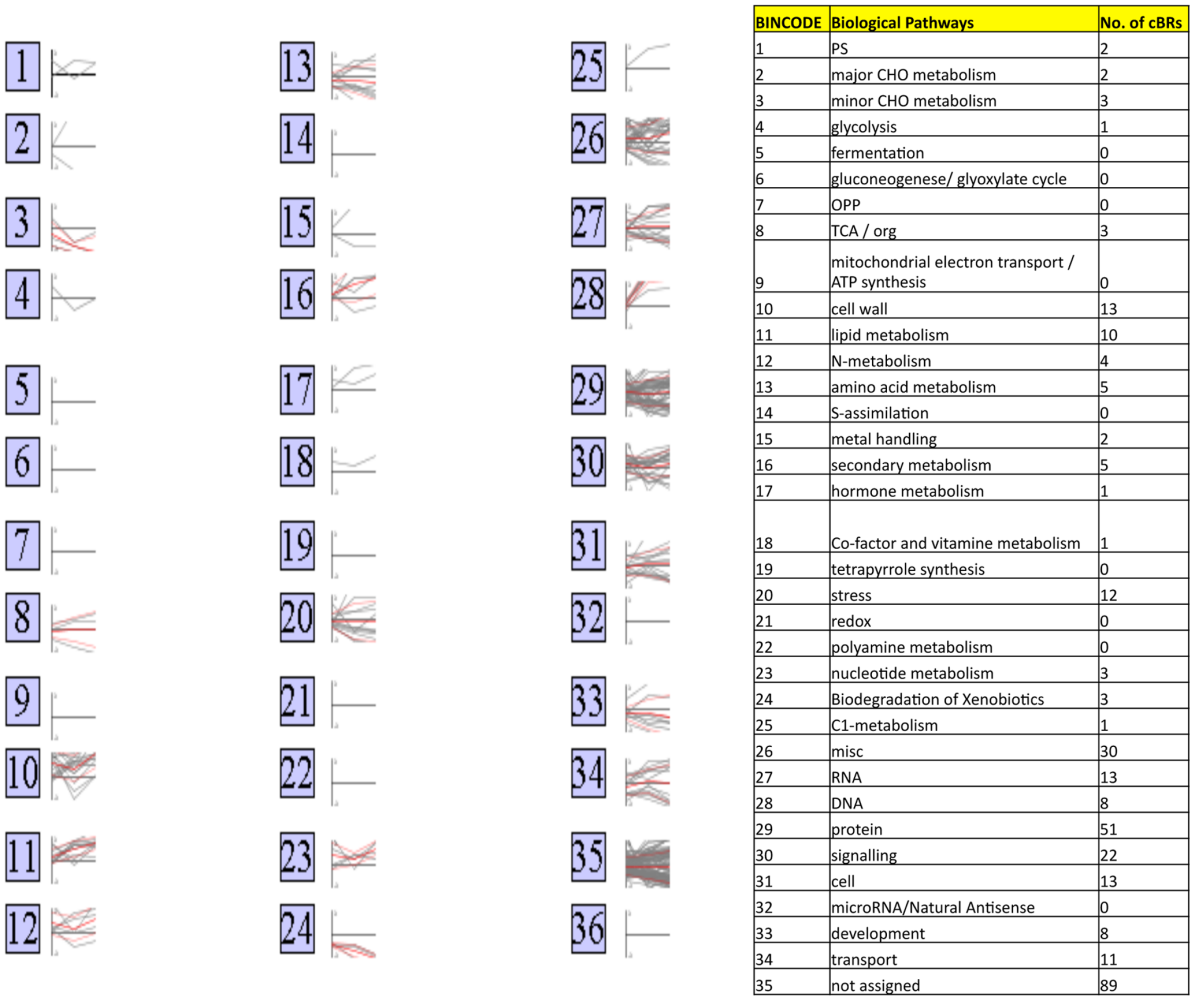
Distribution of cBRs and Their Expression Patterns in Biological Pathways. All cBRs were assigned to 35 functional categories defined by MapMan tools. The log2 of average fold changes from dry seed over the course of germination for each cBR were graphed next to its functional categories. The number of cBRs assigned to each functional group was listed in the table.

The genes represented on the rice and barley GeneChips are classified into 35 functional groups based on their functions in metabolic pathways, signaling pathways and gene families in MapMan and PageMan [24], [25]. The cBRs encoded proteins in most of the functional groups (Figure 2 and Table 3). For examples, 13 cBRs encoded proteins in cell wall metabolic pathways while 22 cBRs were functionally related to signaling pathways. Eighty-nine cBRs encoded proteins that are not classified into any of the functional groups. cBRs in the same functional group often had diverse expression patterns. For example, cBRs in stress-related pathways had both up-regulated and down-regulated expression patterns in early phase of germination. Conversely, cBRs in several functional groups had similar expression patterns. For example, all three cBRs in the biodegradation of xenobioitics pathway were down-regulated in both early and late phases of germination while all eight cBRs except cBR_M23 in DNA related pathways were up-regulated in both early and late phase of germination (Figure 2 and Table 3). Interestingly, a large number of transcription factor genes are differentially regulated over the course of barley germination [9]. However, a limited number of cBRs encoded transcription factors. Only a PHD finger protein (cBR_207) and an AP2/EREBP protein (cBR_191) were down-regulated during seed germination (Table 3). Therefore, germination regulated transcription factor genes evolved quickly in either their protein sequences or/and their expression patterns.

### Biological Pathways Regulated by Conserved Transcriptional Regulatory Programs

**Figure 3:**
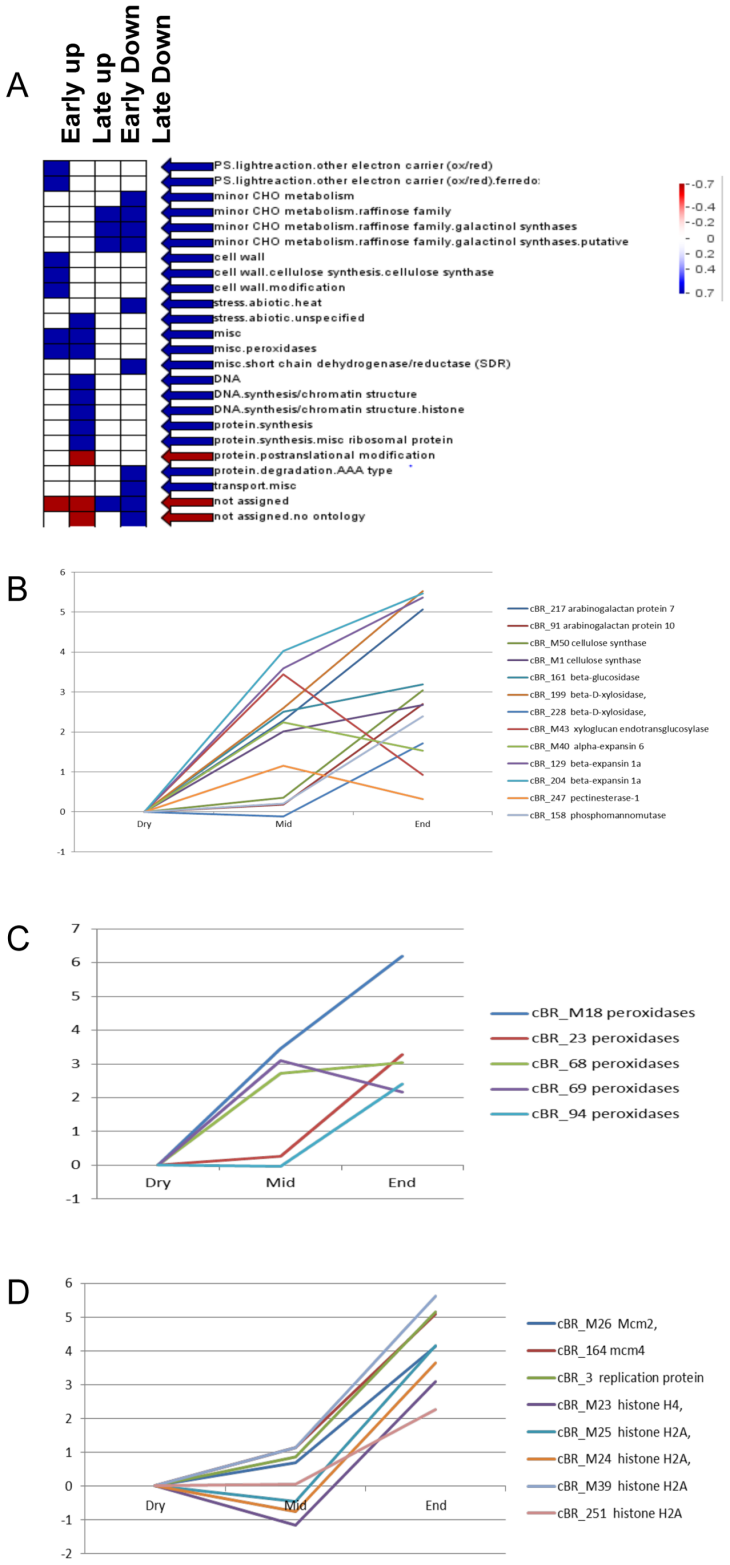
Biological Pathways and Protein Families Over- and Under-Represented with Early and Late Germination Regulated cBRs. Figure 3A showed biological pathways and families over- and under-represented with early or late germination regulated cBRs. The functionalities were displayed on the right; and the germination phase and regulation patterns were displayed on the top. The representation analysis was conducted for all cBRs. Log2 fold change values in early and late germination phases were used in the PageMan analysis. Fisher's exact test and an ORA Cutoff value of 1 were used. A false color scale was used to indicate the statistic Z value. Blue and red indicates significance in over-representation and under-representation. The cBRs encoding proteins in cell wall metabolism and peroxidase families were preferentially regulated in early germination phase (Figure 3B and 3C) while the cBRs encoding proteins in chromatin structure/modeling pathways were preferentially up-regulated in late germination phase (Figure 3D). Log2 of average fold changes from dry seed over the course of germination for the cBRs in those pathways were graphed. Dry, middle (Mid) and end (End) points of germination were indicated as X-axis.

Representation analysis of cBRs in each functional group showed that the cBRs in a number of biological pathways were preferentially regulated in conserved expression programs (Figure 3A). Early germination up-regulated cBRs were over-represented in cell wall metabolic pathways and peroxidase gene family (Figure 3A, 3B and 3C). A total of 13 cBRs such as arabinogalactan protein (AGP), cellulose synthase, beta-glucanase, beta-D-xylosidase, expansins and xyloglucan endotransglucosylase were identified in the cell wall metabolic pathway. All of the 13 cBRs were up-regulated during early germination, except that cBR_228 encoding beta-D-xylosidase was slightly down-regulated (Figure 3B). In addition, five cBRs encoded peroxidases; and four of them were up-regulated in the early germination phase (Figure 3C). Most of the peroxidase genes were also preferentially up-regulated in the late germination phase. It was reported previously that peroxidase activity increases significantly in the micropylar end of germinating tomato seeds [26]. The conserved up-regulation of peroxidase genes in barley and rice provides additional evidence supporting the functional importance of peroxidase in seed germination.

The cBRs encoding chromatin remodeling and structural proteins were preferentially up-regulated during the late germination phase. There were 8 cBRs in chromatin structure pathways. All of them were dramatically up-regulated during the late germination phase by more than 4.7 fold with an average of 30 fold. However, expression levels of those cBRs had no or little change during the early germination phase (Figure 3D). Thus, the specific and strong up-regulation of chromatin-related genes in the late germination phase was conserved in rice and barley. Five of the eight cBRs encoded histone proteins. For example, the cBR_M23 was composed of 8 barley and 2 rice histone H4 genes. Two of the eight cBRs encoding replication licensing factor MCM proteins were specifically up-regulated in late germination phase. MCM encodes a conserved minichromosome maintenance protein and plays an essential function as a helicase in DNA replication elongation in eukaryotes. MCM proteins also participate in other chromosome processes including transcription, chromatin remodeling, and genome stability [27].

### Biological Pathways and Gene Families Containing cRBs with Diverse Expression Patterns

**Figure 4:**
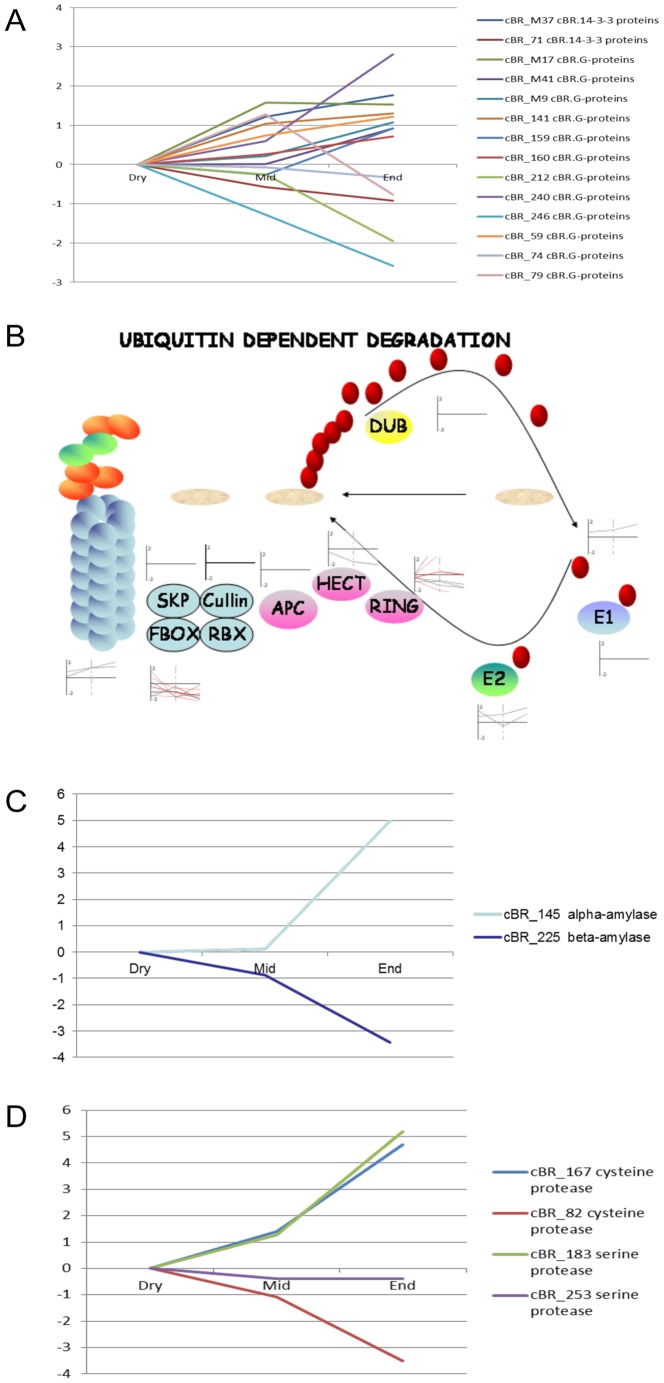
Biological Pathways and Protein Families Composed of cBRs with Divergent Expression Patterns. The cBRs encoding G-proteins and 14-3-3 proteins (4A), proteins in ubiquitin dependent degradation pathways (4B), cysteine and serine proteases (4C), and alpha and beta amylases (4D) with diverse expression patterns were shown. Log2 of average fold changes in reference to dry seeds over the course of germination for each cBR was graphed. Dry, middle (Mid) and end (End) points of germination were indicated X-axis. The diagram of ubiquitin dependent degradation pathway was displayed in 4B.

Interestingly, the cBRs in a number of signaling pathways and gene families had diverse expression patterns. The cBRs encoding 14-3-3 proteins, G-proteins, receptor kinases, calmodulin and calcium-dependent protein kinase in signaling pathways were identified. The expression patterns of those cBRs were highly diverse (Table 3 and Figure 4A). A total of 12 cBRs encoded G-proteins, but their expression patterns were highly diverse over the course of germination. For example, the cBR_M17 was up-regulated by 13-fold in the early germination phase. In contrast, another ras-related G protein cBR (cBR_246) was down-regulated by 2.4 fold in the early germination phase. Two cBRs (cBR-M37 and cBR_71) encoded 14-3-3 proteins. The cBR_71 was down-regulated while cBR-M37 was up-regulated over the course of seed germination. Fourteen cBRs encoded proteins in ubiquitin/26S proteasome-mediated protein degradation pathways, which often play important roles in a variety of signaling transduction pathways (Figure 4B). Most of the cBRs encoded E2 and E3 regulatory proteins such as E2, HECT, RING and F-BOX proteins, and had diverse expression patterns. For example, four cBRs encoding F-box proteins were differentially regulated by seed germination, and showed diverse expression patterns.

Both alpha- and beta-amylases are key enzymes required in seed storage starch mobilization during seed germination and seedling growth [1], [23]. Interestingly, the cBRs encoding alpha- and beta- amylases had opposite transcriptional patterns. The alpha-amylase cBR was up-regulated in late germination stages while the beta-amylase cBR was down-regulated in late germination (Figure 4C). In addition, two cBRs encoding cysteine proteases and two cBRs encoding serine proteases were identified. Both cysteine and serine proteases were suggested to play a role in protein mobilization during seed germination [28]. Interestingly, one cysteine protease cBR and one serine protease cBR were up-regulated while the others were down-regulated in both the early and late germination phase (Figure 4D). The functional and evolutionary significance in preserving the opposite transcriptional regulatory programs for these functionally related genes remains to be explored.

## Discussions

Barley and rice diverged from their common ancestor 50 million years ago [12]. However, they share a great similarity morphologically and physiologically in germination and seedling growth. In this study, we measured the transcriptomes of germinating rice grains at dry, mid- and end points of seed germinations, which should represent the most distinct stages of the dynamic transcriptional changes over seed germination process. Having determined transcriptomes of rice at the three equivalent stages [9], we designed a systems and evolutionary strategy to compare the dynamic transcriptomic changes over the course of seed germination to gain an insight into divergence and conservation of gene regulatory programs underlying rice and barley germination.

One-Way ANOVA analysis of the transcriptomes revealed that 2537 barley and 13813 rice genes were differentially regulated over the course of seed germination. Comparing their encoding protein sequences and expression patterns identified 322 sets of conserved barley and rice genes (cBRs) sharing strong similarity in both protein sequences and gene expression patterns. The collection of cBRs contained 368 barley genes and 388 rice genes. Thus, only a very small percentage of the germination-regulated genes preserved their protein sequences and gene expression patterns; and a significant divergence occurred in transcriptional regulatory programs underlying rice and barley germination since the barley-rice divergence. As expected, protein sequence similarity of germination regulated barley and rice genes positively correlated to the similarity of their expression patterns, suggesting co-evolution of protein functions and gene expression patterns.

Biological functions of genes are mainly determined by their protein sequences and their expression patterns. Both protein sequences and expression patterns change quickly if the genes have no functional significance [17], [29], [30], [31]. Therefore, we hypothesized that the germination regulated expression patterns and protein sequences of the barley and rice genes in each cBR have been preserved for 50 million years after the split of rice and barley from their common ancestor because the genes are functionally important to seed germination, and should contribute to the characteristics shared by rice and barley germination. Additionally, 60 of the 322 cBRs were multi-gene cBRs. Each multi-gene cBRs contained at least one pair of paralogs. Duplicated paralogous genes are subjected to little functional constrains, and offer a great opportunity for their sub-functionalization or neo-functionalization through divergence of their protein sequence and/or expression patterns [17], [19], [20], [21], [32]. Preserving germination regulated expression patterns and protein sequences of those paralogous genes in the multi-gene cBRs suggests that they may be subjected to negative selection, and provides additional evidence supporting their functional significance in seed germination.

We identified a number of biological pathways enriched with cBRs of similar expression patterns, suggesting that their underlying transcriptional regulatory programs are highly conserved in rice and barley. Preserving coordinate regulation of their gene expression patterns across rice and barley in each of those pathways provided further evolutionary evidence for functional significance of those biological pathways in seed germination. As suggested, most of those biological pathways have been previously proposed to functionally important in seed germination based on a variety of evidences. For example, a total of 13 cBRs were identified in cell wall metabolic pathway; and 12 of the 13 cBRs were up-regulated during early germination. Cell wall metabolism plays an important role in germination for most angiosperm seeds. It is required for two important germination biological processes [33], [34], radicle elongation growth and endosperm weakening. It was previously reported that endosperm weakening is accompanied with the induction of cell wall remodeling enzymes in several species. They include endo-beta mannanase, beta-1,3-glucanases, expansins, xyloglucan endotransglycosylase, pectin methylesterase, polygalacturonase and arabinogalactan protein [34]. We identified cBR encoding each of these proteins. Three cBRs encoding expansins were up-regulated during early germination. Expansins are involved in modifying the cell wall matrix during plant growth and development, and have been demonstrated to have cell wall extension activity in vitro and in vivo [35]. It was proposed that expansins is involved in the expansion of cucumber hypocotyls [36]. During germination of tomato seeds, a specific alpha-expansin transcript accumulates in the endosperm cap, presumably in association with the weakening of cell walls that facilitates emergence of the radicle [37]. The functional significance of expansins in germination might be an importance force to preserve the early germination up-regulated expression patterns and protein sequences of the cBRs. Cell wall precursor synthesis, cellulose synthesis and cell call modification genes are up-regulated during the early germination phase in barley [9]. A number of cell wall degradation related genes are preferentially expressed in after-ripening barley coleorhiza, and are likely to associate with breaking seed dormancy [7]. Preserving early germination up-regulation of those cell wall metabolic enzyme genes in barley and rice also provided further evidence supporting the hypothesis that the early germination process turns on the transcriptional regulatory programs underlying cell wall metabolism to weaken coleorhiza and facilitate root emergence.

The cBRs encoding chromatin remodeling and structural proteins were preferentially up-regulated during the late germination phase. There were 8 cBRs in chromatin structure pathways. All of them were dramatically up-regulated during the late germination phase by more than 4.7 fold with an average of 30 fold. Histone modification and chromatin remodeling play important roles in reprogramming transcriptional programs. Chromatin-based regulation of seed dormancy and germination was also reported [38], [39], [40]. Mutation of histone monoubiquitination genes in *Arabidopsis* reduces ubiquitinated forms of histone H2B and alters expression levels for several dormancy-related genes [39]. A transient histone deacetylation event occurs during seed germination one day after imbibition, and is likely to serve as a key developmental signal that affects the repression of a number of histone deacetylase regulated genes [40]. Preserving preferential up-regulated expression of cBRs in late germination phase suggests an important role for histone modification and chromatin remodeling in germination, which likely supports radicle elongation and quick seedling growth in late and post-germination phase.

Interestingly, a number of biological pathways and gene families contained cBRs with diverse expression patterns. The cBRs encoding proteins in signaling pathways such as G-proteins and kinases often had diverse germination regulated expression patterns. G-proteins are involved in seed germination [41]. Diverse expression patterns of those G-protein cBRs suggested that those G-protein cBRs may participate in diverse signaling pathways in seed germination process. Thus, those cBRs had distinct biological functions in the most recent ancestor of barley and rice, and their protein sequences and germination regulated expression patterns have been preserved after their split from the ancestor. In addition, two distinct regulatory programs controlling alpha- and beta- amylases production were conserved in barley and rice. Starch, a major storage reserve in rice and barley grains, is mobilized during seed germination to support seedling growth. Alpha- and beta-amylases are key enzymes required in starch mobilization [1], [23]. The alpha-amylase cBR was up-regulated in late germination stages while the beta-amylase cBR was down-regulated in late germination (Figure 3D). Alpha-amylase genes are up-regulated in cereal grain germination and seedling growth. They are also induced by GA in barley aleurone tissues [10], [15], [23], [42], [43]. Preserving up-regulation of alpha-amylase genes was consistent with its biological functions in starch degradation during seed germination and seedling growth [44]. In contrast, previous biochemical studies showed that beta-amylase is synthesized and stored exclusively in the starchy endosperm during seed maturation rather than in the aleurone after the initiation of germination [45], [46]. Accumulation level of beta-amylase transcript does not respond to GA treatment in barley aleurone [10]. Thus, the alpha- and beta- amylase cBRs had two opposite expression patterns that had been preserved during barley and rice seed germination for 50 million years of barely- rice divergence. Two cBRs encoding protease also showed opposite expression patterns during seed germination. The functional and evolutionary significance in preserving the two opposite transcriptional regulatory programs for these functionally related genes remains to be explored.

We also hypothesized in the study that the barley and rice genes in each cBR have equivalent or similar biological functions because of their strong similarity in protein sequences and expression patterns. Rice serves as a model plant for monocot plant research, and has rich research resources such as a large collection of genetic mutants and substantial genomic information. Barley germination has been extensively studied biochemically and physiologically. Identification of the functionally equivalent rice and barley genes should greatly facilitate integration of research resource and knowledge from rice and barley research. In addition, gene expression changes in response to a biological process are used to successfully predict functional involvement of a gene in the biological process. However, it is often limited to a single species. It is difficult or even impossible to distinguish coincidentally regulated genes from those that are physiologically important. We hypothesized that the evolutionary conservation in the expression patterns of the inter-species and intra-species homologous genes could be used to predict their biological functions with a higher confidence [47], [48]. Overall, the evolutionary and systems strategies described in the manuscript have a broad application in predicting genes functionally important and equivalent in a biological process and translate the research and knowledge across plant species with a great confidence.

## Materials and Methods

### Plant Growth and Harvest

Oryza sativa L. ssp. japonica (cv. Nipponbare) seeds were used in the experiment. Plump and healthy seeds were imbibed in water for three hours and then germinated on water-saturated germination pack in the dark at 30°C. Twenty seeds were planted in each 15 cm diameter Petri dish and spaced evenly to reduce the variation. The seeds at each representative time point of 0 h (dry grains), 21 h and 42 h were harvested. Three replications were conducted for each time point. Each replication represented an independent germination experiment at identical growth condition. The seeds for each replication were pooled together and immediately frozen in liquid nitrogen, and then stored at -80 degree for RNA extraction.

### RNA Purification

Plant tissue (2 g) was ground using a mortar and pestle in liquid nitrogen followed by adding 10 mL extract buffer (4% p-aminosalicylic disodium, 1% 1, 5-naphthalenedisulfonic acid) and 10 ml phenol. The mixture was inverted several times, and then 10 ml chloroform was added; and the solution was homogenized for 45 seconds using a Polytron. After centrifuging, the aqueous phase was transferred into a new tube. Calcofluor white (60 ul of 10% solution) was added, mixed thoroughly and centrifuged for another 15 min at 4°C, 12,000 rpm. RNA in the supernatant was precipitated using 1/10 volume of 3 M NaOAc, and 2 volume of 100% ethanol. After centrifuging, the pellet was dissolved in 8 ml water. 5 ml of 8 M LiCl was added and the solution incubated on ice overnight. The resulting RNA pellet, isolated after centrifugation, was dissolved in water. RNA quality and quantity was determined using a Nano-Drop AN1000 (Nano-Drop, Wilmington, DE) and Agilent 2100 Bioanalyzer (Aglient, Palo Alto, CA).

### Microarray Assay and Data Analyses

Preparation of cDNA and biotin-labeled cRNA were performed and analyzed as recommended by Affymetrix (Santa Clara, CA). According to the manufacturer's protocol, 10 ug of total RNA was used in a reverse transcription reaction to generate first-strand cDNA using SuperScript II (Invitrogen, Clarsbad, CA). After second-stranded synthesis, double-strand cDNA was used for an in vitro transcription reaction to generate biotinylated cRNA. 10 ug of fragmented cRNA for each sample was used in the hybridization. Staining and scanning steps were performed according to the manufacturer's recommended protocols (Affymetrix, Inc., Santa Clara, CA).

The GeneChip probe-level data were background-corrected, normalized and summarized based on GC-Robust Multi-Array Analysis (RMA) approach [49]. In this approach, quantile normalization was used to remove the variation introduced during sample preparation, manufacturing of the arrays, and the processing of arrays, so that GeneChips from different time points and replicates are comparable, and expression level value for each gene was derived from probe pairs based on a log scale linear additive model [50].

Then pre-normalized data were analyzed with Genespring 7.2 software (Silicon Genetics, Redwood City, CA). Within each array, a further “per gene normalize the median” (with cutoff 0.01) was applied. The most unreliable data with absent call across 9 chips based on analyzed result using Microarray Suite 5.0 (Affymetrix, Santa Clara, CA) were filtered out. Statistical analyses were performed using a one-way ANOVA provided in GeneSpring 7.2 software (With Parametric Test, Variances Assumed Equal Option; Benjamini and Hochberg multiple testing correction. FDR set at 0.05) to identify genes that were differentially expressed among samples at any two time points during seed germination.

Considering that the potential non-specific hybridization between homologous genes could lead to cause an inaccurate correlation of their expression profiles, we excluded probes flagged by Affymetrix as potentially cross-hybridizing. The flagged probe sets included the ones with _x _at, which designates probe sets where it was not possible to select either a unique probe set or a probe set with identical probes among multiple transcripts, _s _at, which designates probe sets with common probes among multiple transcripts from different genes and _i_at, _g_at, _f_at, _r_at.

### Identification of Barley-Rice (BR) Genes

The exemplar sequences of all probe-sets on Barley Genome GeneChip and Rice genome GeneChip were downloaded for the GeneChips used (http://www.affymetrix.com/products/arrays). An all-against-all reciprocal tBLASTX search was used to identify BRs at a given sequence homology. Pearson correlation coefficients (PCCs) of log_2_ expression values were calculated between homologs in R. Barley and rice genes with significantly changed expression level during seed germination were permuted to produce 100,000 random pairs to determine the distribution of PCCs for the randomized population. Chi-square analysis was used for comparison of observed values between barley and rice genes in each BR and PCCs values from randomized pairs. Chi-square analysis was used for comparison of expression values between observed and random pairs. The microarray data used in the studies were deposed in NCBI Gene Expression Omnibus database (GSE 23595).
